# Mice Lacking NCF1 Exhibit Reduced Growth of Implanted Melanoma and Carcinoma Tumors

**DOI:** 10.1371/journal.pone.0084148

**Published:** 2013-12-16

**Authors:** Tiina Kelkka, Angela Pizzolla, Juha Petteri Laurila, Tomas Friman, Renata Gustafsson, Eva Källberg, Olof Olsson, Tomas Leanderson, Kristofer Rubin, Marko Salmi, Sirpa Jalkanen, Rikard Holmdahl

**Affiliations:** 1 Medicity Research Laboratory, Turku, Finland; 2 Turku Doctoral Programme of Biomedical Sciences, Turku, Finland; 3 Medical Inflammation Research, Department of Medical Biochemistry and Biophysics, Karolinska Institutet, Stockholm, Sweden; 4 Department of Medical Biochemistry and Microbiology, Uppsala University, Uppsala; 5 Immunology Group, Biomedical Center, Lund University, Lund, Sweden; IDI, Istituto Dermopatico dell'Immacolata, Italy

## Abstract

The NADPH oxidase 2 (NOX2) complex is a professional producer of reactive oxygen species (ROS) and is mainly expressed in phagocytes. While the activity of the NOX2 complex is essential for immunity against pathogens and protection against autoimmunity, its role in the development of malignant tumors remains unclear. We compared wild type and *Ncf1* ^*m1J*^ mutated mice, which lack functional NOX2 complex, in four different tumor models. *Ncf1* ^*m1J*^ mutated mice developed significantly smaller tumors in two melanoma models in which B16 melanoma cells expressing a hematopoietic growth factor FLT3L or luciferase reporter were used. *Ncf1* ^*m1J*^ mutated mice developed significantly fewer Lewis Lung Carcinoma (LLC) tumors, but the tumors that did develop, grew at a pace that was similar to the wild type mice. In the spontaneously arising prostate carcinoma model (TRAMP), tumor growth was not affected. The lack of ROS-mediated protection against tumor growth was associated with increased production of immunity-associated cytokines. A significant increase in Th2 associated cytokines was observed in the LLC model. Our present data show that ROS regulate rejection of the antigenic B16-luc and LLC tumors, whereas the data do not support a role for ROS in growth of intrinsically generated tumors.

## Introduction

Reactive oxygen species (ROS) are instrumental in defense against bacterial and fungal infections and also serve as important regulators of inflammation and immunity [1,2]. The most important immunological sources of ROS are phagocytes, whose NADPH oxidase 2 (NOX2) complex produces massive amounts of ROS upon activation. ROS produced by antigen presenting cells downregulate T cell activity, thereby reducing the severity of autoimmune diseases [2,3]. 

The effects of ROS on tumor growth have been widely studied and ROS have been shown to both suppress and support tumor growth [4]. However, in many studies focusing on the role of ROS in malignant diseases, the source of the ROS has not been specified. Recently, NOX family oxidases were shown to promote cell proliferation in acute myeloid leukemia [5]. Furthermore, the expression levels of NOX1 [6], NOX5 [7], as well as the related dual-oxidases DUOX1 and DUOX2 [8] have been shown to associate with tumor development. ROS derived from the NOX2 complex have been suggested to support the survival of human leukemia cells [9] by suppressing the anti-tumor T and NK cell responses [10] and furthermore, histamine that blocks ROS production in monocytes/macrophages improves survival in metastasizing melanoma when used as an adjunct therapy to IL-2 [11]. Experiments using siRNA technology have shown that NOX4 mediates renal cell carcinoma invasion [12], further supporting the tumor-promoting role of NOX family enzymes.

Studies addressing the role of ROS in cancer development are commonly performed by either detecting the expression levels of the oxidase of interest from various tumors or by using chemical ROS scavengers and/or inhibitors. Increased gene/protein expression may be a secondary effect reflecting the metabolic changes in the transformed cell and chemical ablators of ROS similarly as pharmacological inhibitors of the NOX family members may lead to off-target effects. Thus, good *in vivo* models are warranted to elucidate the role of different radicals and different cellular sources of ROS. 

Our goal was to study the role of NOX2 complex derived ROS on tumor growth *in vivo* by using a validated mouse model that specifically lacks the function of the NOX2 complex. The role of the NOX2 complex in the propagation of solid tumors has not been addressed in the literature. 

The development of tumors can be studied *in vivo* by using animal models. Malignant tumors are either induced by transplantation of tumorigenic cells or by germ line targeted genetic modifications that induce spontaneous tumor growth. B16 melanoma [13] and Lewis lung carcinoma (LLC) [14], both derived from C57/BL6 mice, are well-characterized models that are induced by engrafting *in vitro* propagated cells to recipient mice. As a model for spontaneous carcinoma, we used the Transgenic Adenocarcinoma of the Mouse Prostate (TRAMP) mouse [15]. These models were used to assess the impact of phagocyte ROS on tumor development and anti-tumor immunity. NOX2 complex derived ROS was found to support tumor growth in B16 melanoma and LLC models. Furthermore, we provide evidence that NOX2 complex derived ROS support tumor growth via an immunological pathway. 

## Materials and Methods

### Mice

Mice with the *Ncf1* ^*m1J*^ mutation (protein also called p47phox) (The Jackson Laboratory, Bar Harbor, Maine) were backcrossed for more than 10 generations onto the C57BL/10.Q/rhd (B10.Q) background as previously described [3] and checked for genetic purity ascertaining that the mice only differ by the mutation in the *Ncf1* gene. 

### Ethics statement

All of the experimental procedures were in accordance with European union guidelines on animal welfare. The animal experiments were approved by the National Animal Experiment Board in Finland (ELLA) with permit number ESLH-2008-03700/Ym-23, the ethical committees for animal experiments in Uppsala, Sweden (permit number: C76/10) and in Lund, Sweden. 

### B16 Melanoma and Bioluminescence Imaging

The production of the FLT3L expressing B16 melanoma cells (B16-FLT3L) was described in [16]. These cells were a gift from Prof. Granucci and were cultured as described in [16] and injected (5 x 10^6^ per mouse) subcutaneously (s.c.) in the right shoulders of wild type (*Ncf1* ^*+/+*^) and *Ncf1* ^*m1J*^ mutated (*Ncf1* ^*/*^) mice. The experiments using luciferase expressing B16-F10-luc-G5 cells (briefly B16-luc) (Xenogen) were performed as described in [13] and *in vivo* imaged as described in [17].

### Flow Cytometry

After hypotonic lysis of the red blood cells, spleen cells from the B16-FLT3L tumor bearing mice were enumerated using a haemocytometer and stained with antibodies against CD11c, Gr-1, B220, Ly6g (Becton Dickinson), CD11b and CD3 (eBioscience). Different cell populations were phenotypically gated from all splenocytes/ whole blood, as given in figure legends.

For the analysis of oxidative burst, leukocytes were suspended in Hank’s balanced salt solution and incubated for 10 minutes at 37°C with 3µM dihydro-rhodamine 123 (DHR-123; Molecular Probes and Invitrogen Life Technologies) followed by 20 minutes incubation with 200 ng/ml Phorbol 12-Myristate 13-Acetate (PMA, Sigma-Aldhrich) or 3 hours incubation with 1mg/ml zymosan (Sigma-Aldhrich) at 37°C. 

Tumor-infiltrating leukocytes were isolated from minced tumor pieces after collagenase D digestion (1 mg/mL, +37°C, 40 minutes) and treated with DNase I. The cells were stained with CD45-APC-Cy7, CD11b-PB, Ly6c-FITC, Gr-1-PE (Becton Dickinson) and F4/80-APC antibodies and treated with 7-aminoactinomycin D (7-AAD) to assess cell viability. 

The cells were acquired on FACS Calibur with CellQuest software or LSR II equipped with FACS Diva software (BD Biosciences). Data were analyzed with Flowing Software (Cell Imaging Core, University of Turku, Finland) or by using FlowJo version 8.8.6 (Tree Star Inc. Ashland, OR).

### Lewis Lung Carcinoma

Lewis Lung Carcinoma (LLC) cells (ATCC, Manassas, USA) (150,000 cells / mouse) were injected subcutaneously in the left flanks of *Ncf1* ^*+/+*^ and *Ncf1* ^*/*^ mice in 50 µl PBS. LLC cells were maintained in DMEM (Life Technologies) supplemented with penicillin and streptomycin (SVA, Uppsala, Sweden) in 10 % FBS (Saveen-Werner, Limhamn, Sweden) at 37°C and 5 % CO_2_ in a humidified incubator. For tumor take experiments the mice were monitored for 44 days after inoculation. Tumor dimensions were measured with a caliper and the volumes were calculated with the formula: π/6×height×length×width. A tumor was defined as a subcutaneous mass that grew over time. Mice with tumor volumes >500 µl were anaesthetized with isoflurane (Abbot, Solna, Sweden) and the tumor interstitial fluid pressure (IFP) was measured with the wick in the needle technique as previously described [18]. 

### RNA Extraction and Quantitative Real Time PCR (qRT-PCR)

Total RNA was extracted from tumors with RNeasy Mini Kit (Qiagen, Valencia, CA) according to the manufacturer’s instructions and treated with DNase I (Qiagen) for 20 minutes in room temperature. RNA integrity was verified with electrophoresis on 2% agarose gels. One μg total RNA was subjected to first strand cDNA synthesis with SuperScript® VILO™ cDNA Synthesis Kit (Invitrogen, San Diego, CA). QRT-PCR was performed on LightCycler (Roche Applied Science, Germany) by mixing cDNA with Maxima™ SYBR Green qPCR Master Mix (Fermentas, Germany) and gene specific primers (sequences available upon request). Amplification results were analyzed using LightCycler software Version 3. The calculated threshold cycle values for each gene were normalized to the threshold cycle value of the internal standard *Hprt1* or *Gapdh*. Relative gene expression levels in tumors from *Ncf1* ^*m1J*^ mutated mice were normalized to the average gene expression level in tumors from wild type mice using the 2 - ddCt method [19]. Results were plotted as fold change in gene expression in tumors collected from *Ncf1* ^*m1J*^ mutated vs. wild type mice (the gene expression value in wild type tumors was set to 1). 

### TRAMP Prostate Cancer Model

Female C57Bl/6 TRAMP mice [15] that were heterozygous for the Probasin SV-40 Tag transgene were bred to non-transgenic C57Bl/6 males (Taconic M&B, Ry, Denmark), as well as to *Ncf1* ^*m1j*^ mutated (*Ncf1* ^*/*^) mice on a C57Bl/6 background (The Jackson Laboratory, Bar Harbor, Maine). Littermate controls were used for all experiments. TRAMP tumors were scored by blinded palpation and sacrificed upon being palpation positive. Necropsy was performed on all animals to confirm the palpation result and only animals that had a visible prostate tumor to the naked eye were scored as being tumor bearing. 

### Statistics

Statistical analyses were performed using GraphPad Prism 4, Sigma Plot or SPSS software. Mann Whitney U -test was used to analyze differences between two groups with unequal variance and Student’s t-test was used for sample groups with equal variance. For tumor take, Chi square -test was used to evaluate intergroup differences. The survival curves are displayed as Kaplan–Meier curves and the log rank-test was used to assess the statistical significance. All p-values < 0.05 were considered as statistically significant. 

## Results

### Ncf1 Mutated Mice Developed Smaller B16-FLT3L Tumors

**Figure 1:**
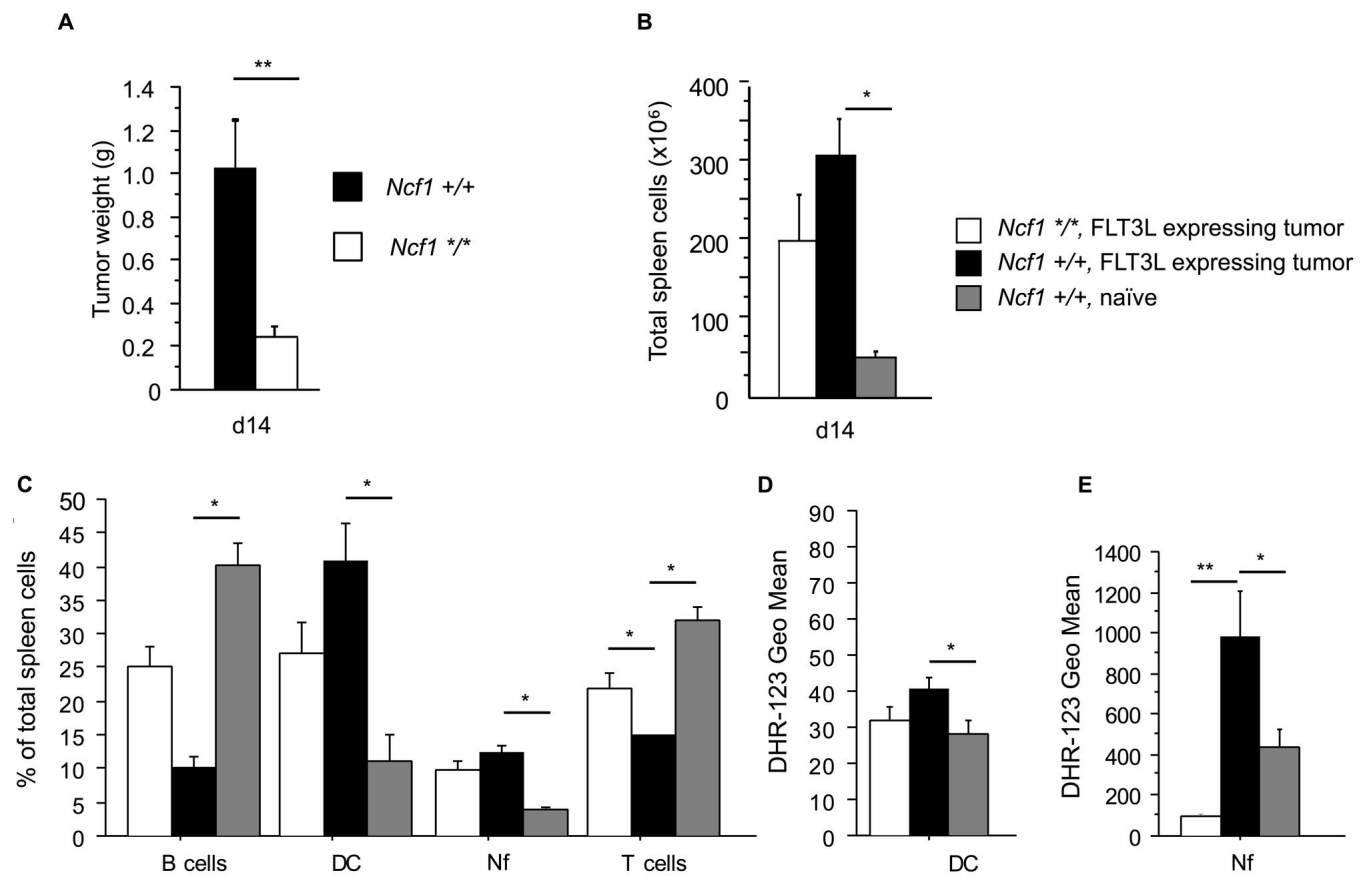
The Development of the B16-FLT3L tumors is restricted in the absence of functional NOX2 complex. **A**) The weights of the B16-FLT3L tumors in *Ncf1* ^*m1J*^ mutated (Ncf1 */*, n=7) and wild type (*Ncf1 +/+*, n=5) mice. All data presented in Figure 1 was collected d14 after tumor implantation. **B**) The total number of spleen cells in the *Ncf1* ^*m1J*^ mutated (Ncf1*/*) and wild type (Ncf1 +/+) B16-FLT3L tumor bearing (FLT3L expressing tumor) and in naïve wild type mice (*Ncf1 +/+*, naïve). **C**) The percentages of B cells (B220+), dendritic cells (DC, CD11c-HIGH) neutrophils (Nf, Gr-1+) and T cells (CD3+) were gated from all splenocytes. Oxidative burst in splenic **D**) DC (CD11c-HIGH) and **E**) Nf (Gr-1+). The studied cell populations were phenotypically gated from all splenocytes. n=5 in all groups, bars represent mean values ±SEM, Mann-Whitney U test, p<0.05 is denoted with * and p<0.01 with **.

FLT3L-expressing B16 tumors were inoculated subcutaneously in wild type B10.Q mice to expand the dendritic cell (DC) population. To our surprise, tumor growth was significantly reduced in the *B10.Q.Ncf1* ^*m1J*^ mice that lack functional NOX2 complex (Figure 1A). The tumors expressing the hematopoietic growth factor FLT3L increased spleen cellularity in both genotypes (Figure 1B) by expanding the DC and neutrophil populations (Figure 1C). Splenic DC (Figure 1D) and neutrophils (Figure 1E), from B16-FLT3L tumor bearing mice had elevated PMA stimulated oxidative burst when compared to naïve wild type mice. The FLT3L inoculated *B10.Q.Ncf1* ^*m1J*^ mutated mice were confirmed not to produce ROS after PMA stimulation (Figure 1D and E)

The B10.Q.*Ncf1* ^*m1J*^ mutated mouse differs from the B10.Q mouse by only one single nucleotide mutation leading to a non-functional NCF1 protein and completely abolished NOX2 production of reactive oxygen species (ROS). Thus, it seems that the presence of NOX2 complex derived ROS has an important function in tumor promotion. 

### Ncf1 Mutated Mice Developed Smaller B16-luc Tumors

To examine if restricted tumor growth in the *Ncf1* ^*m1J*^ mutated mice was due to a FLT3L dependent mechanism, we repeated the experiment with B16-luc cells. To avoid subjective measurement bias, we performed the following experiments using B16-luc cells that express luciferase and allow *in vivo* –imaging based evaluation of tumor growth. The luciferase insert has been shown not to affect tumor cell growth under *in vivo* conditions [20]. 

**Figure 2:**
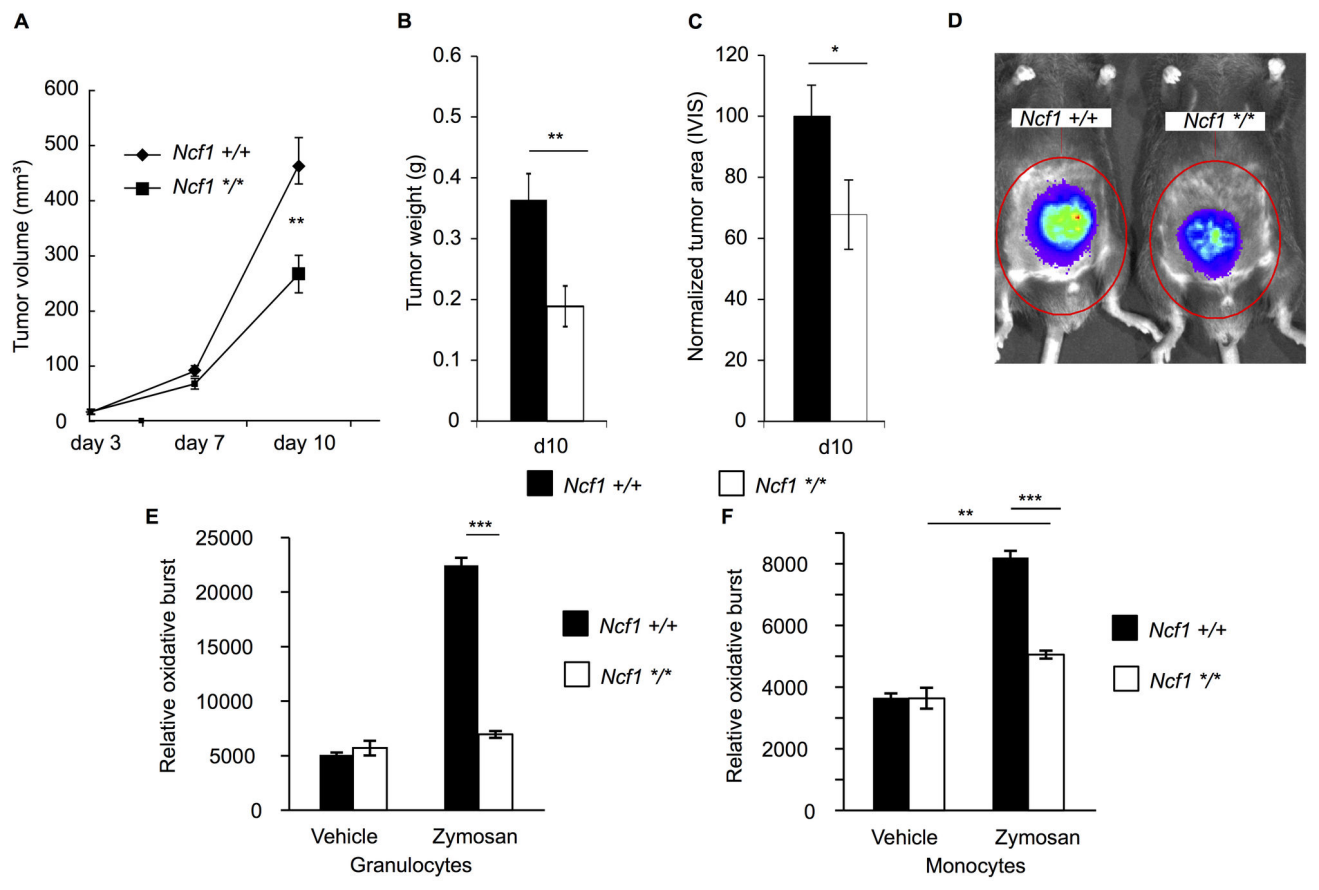
The Development of B16-luc tumors is restricted in the absence of functional NOX2 complex. **A**) The B16-luc tumor volumes in *Ncf1* ^*m1J*^ mutated (Ncf1 */*, n=24) and wild type (*Ncf1 +/+*, n=25) mice. **B**) Tumor weight at termination d10 (Ncf1 */*, n=9, *Ncf1 +/+* n=9). **C**) Bioluminescence imaging together with digital image analysis was used to calculate the tumor area (n=10 in both genotypes), Student’s t-test. **D**) Bioluminescence signal illustrates the difference between the *Ncf1* ^*m1J*^ mutated and wild type mice. **E**) Phagocytosis-induced production of ROS after 3 hours in the incubation buffer (vehicle) or with zymosan in granulocytes (CD11b+Ly6g+) and in **F**) monocytes (CD11b+Ly6g-). The studied cell populations were phenotypically gated from all splenocytes. n=6 in *Ncf1+/+* and n=4 in Ncf1 */*, Mann Whitney U test. Bars represent mean values ±SEM, p<0.05 is denoted with *, p<0.01 is denoted with ** and p<0.001 is denoted with ***.

After the initial phase with an identical growth rate, the *Ncf1* ^*m1J*^ mutation restrained B16-luc tumor growth and the difference in tumor volume grew significant by day 10 (Figure 2A) resulting in 42.6 % smaller tumor volume and 48 % lower tumor weight (Figure 2B) in the *Ncf1* ^*m1J*^ mutated mice. The largest tumors developed a necrotic core and thus, bioluminescence intensity could not be directly used to measure tumor growth. However, using digital image analysis, we could analyze the tumor area and found significantly smaller tumor areas in the mutated mice (Figure 2C), as visually demonstrated in Figure 2D.

Peripheral blood leukocytes were incubated for 3 hours with zymosan and stained for intracellular ROS production. Both granulocytes (CD11b+ and Ly6g+) (Figure 2E) and monocytes (CD11b+ and Ly6g-) (Figure 2F) from the wild type mice exhibited significant ROS production, while granulocytes from the *Ncf1* ^*m1J*^ mutated mice did not produce any ROS. 

We conclude that lack of NOX2 produced ROS protect against B16 melanoma, and is not dependent on FLT3L expression.

### Characterization of the Inflammatory Response against B16-luc Melanoma in Ncf1 Mutated and Wild Type Mice

**Figure 3:**
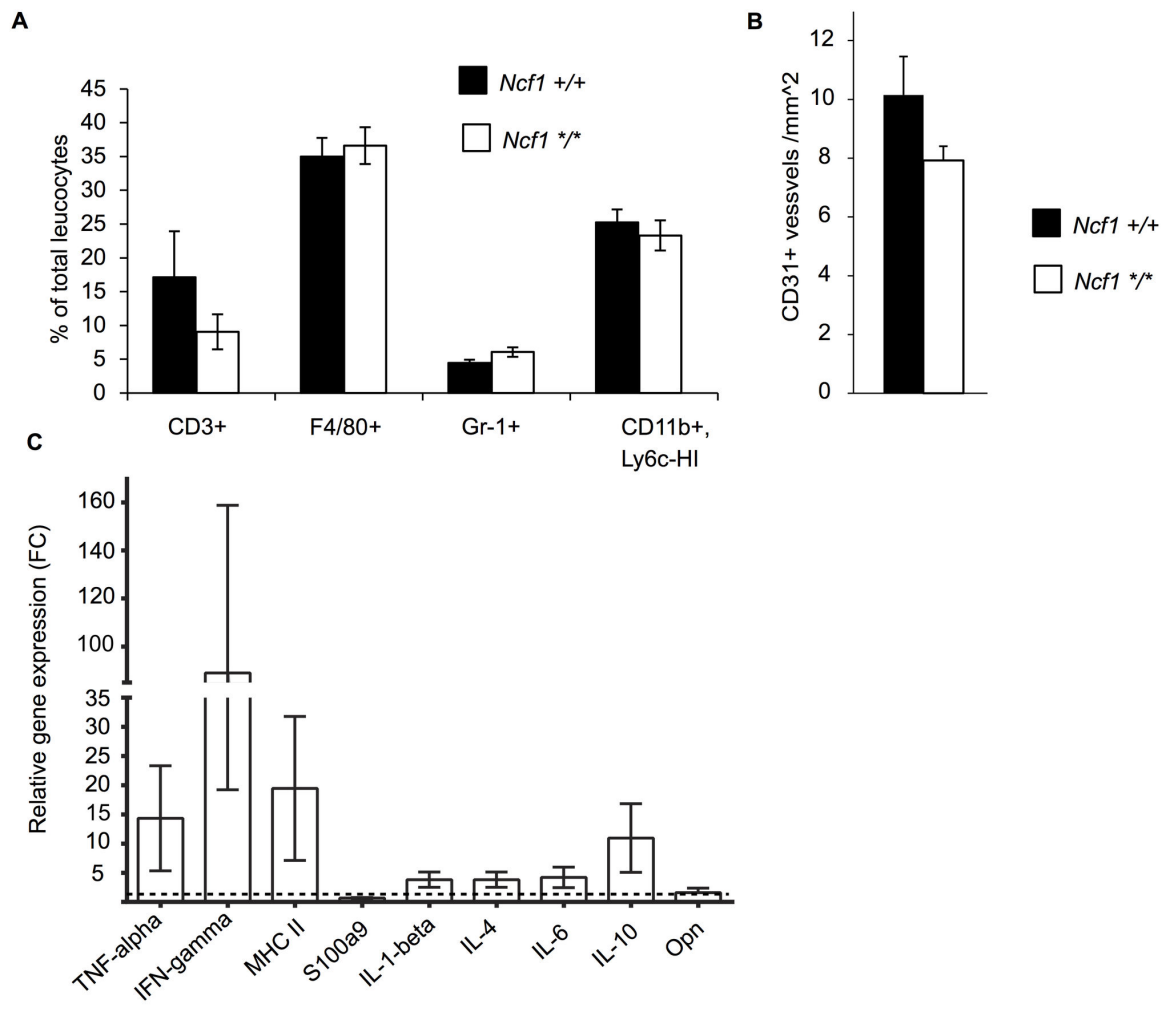
Immune parameters and angiogenesis in B16-luc tumors. **A**) Tumor infiltrating leukocytes in B16-luc tumors at day 10 were analyzed by flow cytometry (n=10 in *Ncf1 +/+* and n=9 in Ncf1 */*). The CD45+ leukocytes were divided into lymphocytes (CD3+), macrophages (F4/80+), granulocytes (Gr-1+) and in suppressor monocytes (CD11b+, Ly6c-HIGH). **B**) The CD31+ vessels were enumerated in tumor tissue sections and presented as number of vessels per square millimeter. **C**) The B16-luc melanoma tumors (n=5) were collected from the *Ncf1* ^*m1J*^ mutated and wild type mice and the tumors were subjected to qRT-PCR analysis of inflammatory cytokines. The average gene expression level in the tumors collected from the wild type mice was set to 1 and the results are presented as fold change (FC) relative to the wild type. Bars represent mean values ±SEM, Student’s t-test.

Next, we analyzed the proportions of T-lymphocytes (CD3+), macrophages (F4/80+), granulocytes (Gr-1+) and suppressor monocytes (CD11b+ and Ly6c-HI) in the B16-luc tumors without finding differences between the genotypes (Figure 3A). To analyze neo-angiogenesis, cryo-sections were stained with anti-CD31. There was no difference in the vascular density in B16 melanoma tumors collected from *Ncf1* ^*m1J*^ mutated and wild type mice. (Figure 3B). 

To further study the mechanism of tumor suppression in the absence of functional NOX2 complex, the expression levels of inflammatory cytokines were analyzed. The expression levels of proinflammatory cytokines were higher in the tumor-protected *Ncf1* ^*m1J*^ mutated mice, in particular TNF-α and IFN-γ, although this did not reach statistical significance. (Figure 3C). Thus, in spite of the clear effect on tumor size we could not find any other striking parameter, except that the lack of ROS possibly enhanced the inflammatory response.

### The Incidence of LLC Lung Carcinoma was lower in the Ncf1^*m1J*^ Mutated Mice

**Figure 4:**
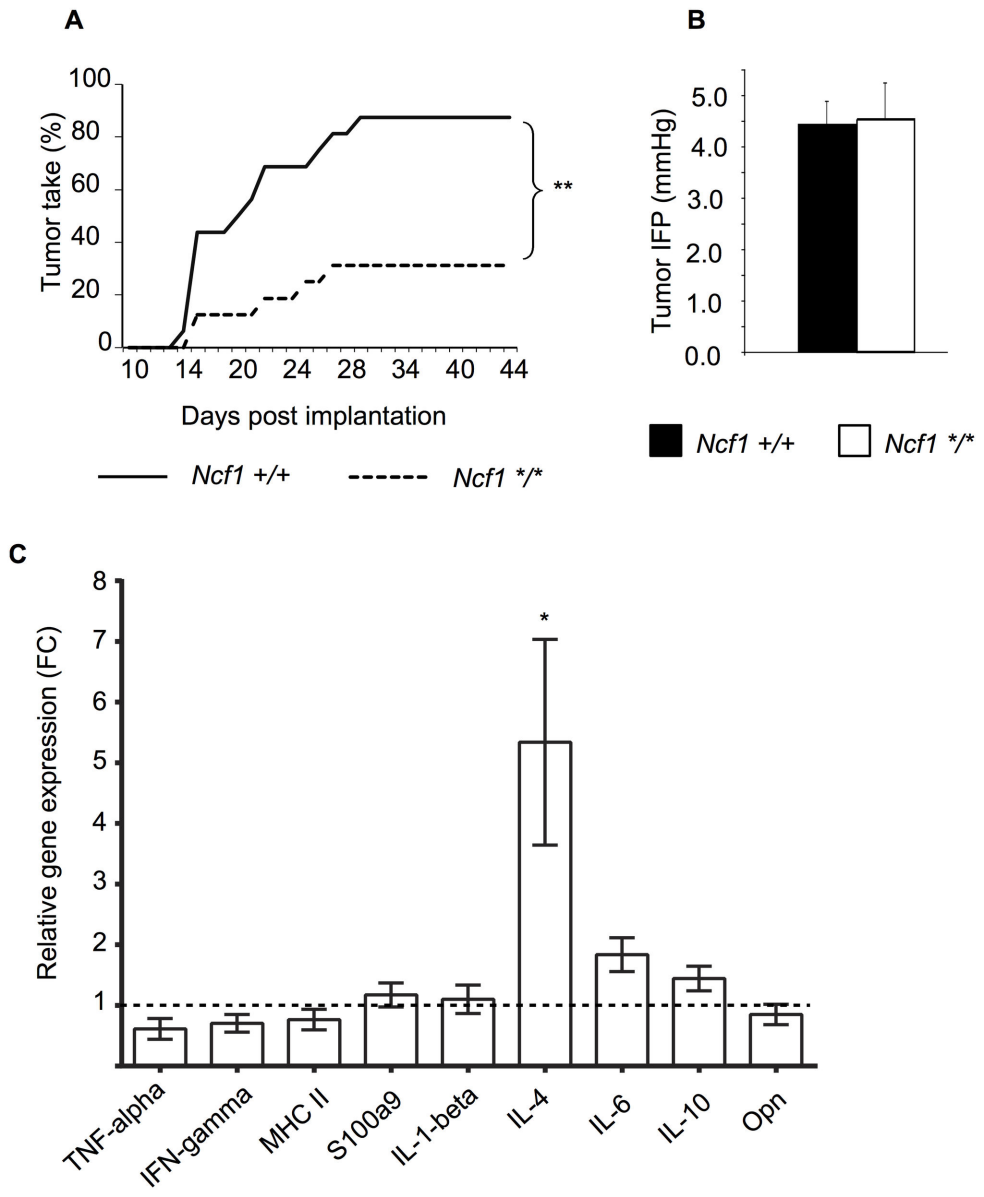
The development of LLC tumors is restricted in the absence of functional NOX2 complex. **A**) The incidence of Lewis lung carcinoma (LLC) tumors in *Ncf1* ^*m1J*^ mutated (Ncf1*/*, n=16) and wild type (*Ncf1+/+*, n=16) mice, Chi square test. **B**) Interstitial fluid pressure (IFP) was measured in the *Ncf1* ^*m1J*^ mutated (Ncf1*/*, n=9) and wild type (*Ncf1+/+*, n=9) mice, Student’s t-test. **C**) LLC carcinoma tumors (n=5) were collected from *Ncf1* ^*m1J*^ mutated and wild type mice and the tumors were subjected to qRT-PCR analysis of inflammatory cytokines. The average gene expression level in the tumors collected from the wild type mice was set to 1 and the results are presented as fold change (FC) relative to the wild type. Bars represent mean values ±SEM, Student’s t-test, p<0.05 is denoted with *.

To investigate whether the ROS effect was unique to melanomas, we continued by studying the well-known LLC carcinoma model. The fraction of *Ncf1* ^*m1J*^ mutant mice that developed measurable LLC tumors was significantly lower when compared to the wild type mice (31% vs. 88 %; Figure 4A). However, the average latency period before measurable tumors were detected was similar in both *Ncf1* ^*m1J*^ mutated and wild type mice (21.6 vs. 20 days, p=0.5). 

Elevated Interstitial fluid pressure (IFP) is a characteristic of carcinoma [21] and to better characterize the difference in tumor take in the LLC model, IFP measurements were performed for tumors of equal size (approximately 500 μl). There was no difference in the IFP between the *Ncf1* ^*m1J*^ mutated and wild type mice (Figure 4B). 

However, analyzing the cytokine expression in a comparable way as with the B16 tumors we found that the IL-4 response was significantly increased in *Ncf1* ^*m1J*^ mutated mice with LLC tumors as compared to the wild type B10.Q mice with LLC (Figure 4C). 

### The *Ncf1* ^*m1J*^ mutation did not affect the tumor free survival in the TRAMP prostate carcinoma model

**Figure 5:**
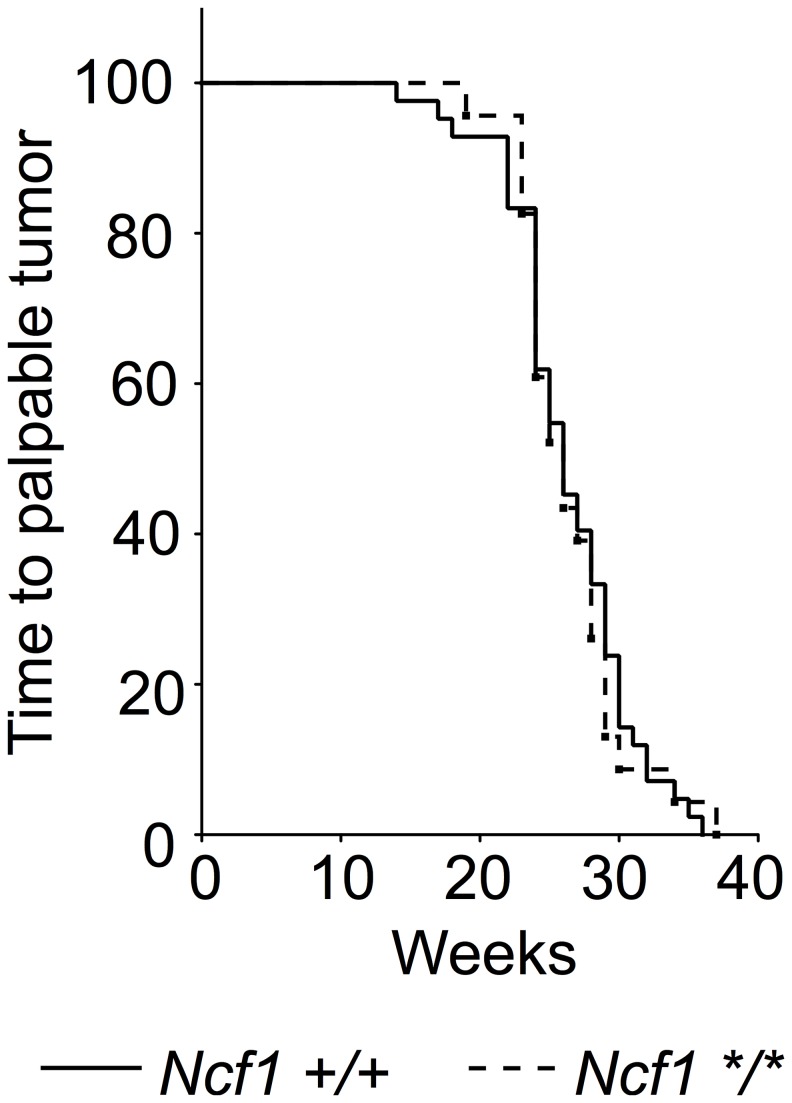
Survival of TRAMP mice with (Ncf1 */*, n=23) and without (*Ncf1 +/+*, n=42) *Ncf1* ^*m1J*^ mutation displayed as Kaplan-Meier curve, log rank test.

To assess the role of the NOX2 complex in a spontaneous tumor model, which is syngenic and develop without immune rejection, the *Ncf1* ^*m1J*^ mutated mouse was introgressed into TRAMP transgenic C57Bl/6 mice. Tumor formation was followed by palpation and all palpation positive mice were sacrificed to visually confirm the presence of a tumor. *Ncf1* ^*m1J*^ mutation did not affect tumor development rate in the TRAMP model (Figure 5). The median for tumor-free survival for both genotypes was 26 weeks.

## Discussion

Using a genetically defined *Ncf1* ^*m1J*^ mutated mouse strain we have addressed whether ROS influence tumor growth in commonly used mouse models. NOX2 complex derived ROS was found to support tumor growth in B16 melanoma, and LLC lung cancer model confirmed this finding. In contrast to the two explanted tumor models, NOX2 complex derived ROS did not affect the length of the tumor free survival period in the spontaneous TRAMP prostate cancer model. 

Our interest for the role of ROS in tumor development stems from discoveries in the field of autoimmunity. When determining the genes controlling chronic inflammation we have found that the most important naturally selected polymorphism enhancing autoimmune arthritis in rats was a deficient *Ncf1* allele [22]. This discovery was reproduced in mouse [3] and the same mechanism seems to operate also in humans [23-25]. This was surprising, as it was generally believed that ROS promotes inflammation. The most likely explanation is that NOX2 derived ROS are important regulators of inflammation (reviewed in 2). However, earlier results using *in vitro*-approaches and knockout mice pointed towards other directions. Thus, it is important to utilize naturally selected polymorphisms and genetically controlled mouse strains.

Using the same genetically controlled mice as previously used for investigating autoimmunity, we now show that the selected transplanted allogeneic tumor models developed more vigorously in the presence of ROS. B16-luc melanoma and LLC models were chosen to represent two very different types of cancer. Possibly, this could be due to similar regulatory mechanisms as operating in autoimmune arthritis. Interestingly, this was only observed in tumor models that were transplanted, and contained allogeneic differences with the host in the MHC region. Contrastingly, ROS did not affect the occurrence of the spontaneously developing TRAMP prostate cancer in which no allogeneic differences and possibly no significant immune mediated rejection against neo-epitopes occurred that could protect the mice. 

The aim of this communication was not to clarify the mechanism for the observed ROS protective effect although several observations point towards a regulatory effect mediated by the host inflammatory response. In the LLC carcinoma model, but not in the B16 melanoma model, the proinflammatory gene expression profile in the tumors collected from the ROS deficient mice was dominated by IL-4. B16-luc tumors grew at a slower pace in the *Ncf1* mutated mouse than in the wild type controls. Interestingly LLC growth was similar in both genotypes, while significantly fewer mutated mice even started to develop a palpable tumor. The expression levels of inflammatory genes were measured only from the tumors that actually developed, thus leading to unavoidable selection bias that also may explain the differences between the two tumor models.

ROS deficient mice are more susceptible to various arthritis models, regardless of the model is mediated by a TH1 (with IFN-γ), TH17 (with IL-17 and IL-23) or even a TH2/IL-13 (with IL-4 and IL-13) type of immune response. Interestingly, in an adjuvant-free arthritis model an increased IL-4 and IL-5 production was associated with eosinophil infiltration in ROS deficient mice [26]. 

Furthermore, ROS seems to raise the activation threshold of autoreactive T cells and also to downregulate the activity of inflammatory macrophages (reviewed in 2). Possibly, similar mechanisms may operate in the rejection of the transplanted tumors leading to altered activation of the allogeneic tumor infiltrating T cells. Tumor-induced myeloid-derived suppressor cells (MDSC) are another set of immune cells with immunosuppressive function and they suppress CD8+ T cell mediated antitumor responses. This suppression is antigen dependent and has been suggested to operate via a ROS dependent mechanism [27]. Recently, chemical ablation of ROS was shown to suppress tumor development by suppressing the differentiation of the suppressive tumor associated macrophages [28]. Our research using a carefully characterized mouse model with genetic ROS deficiency supports the data and suggest that the *in vivo* phenotypes presented by Zhang et al. may be linked to the phagocyte NOX2 complex. Mechanistically, the immune suppression that supports tumor development may even be mediated by physical disruption of the TCR complexes leading to inhibition of T cell activation [29] and thereby altering T cell mediated tumor rejection.

The present observation adds to the view that ROS are important regulators of chronic inflammation, of importance not only in autoimmune diseases but also in the selected transplanted melanoma and carcinoma tumors. The question whether ROS play a role in regulating spontaneously arising tumors remains, however, to be investigated. Possibly, the TRAMP tumor is an exception and if other spontaneously arising tumors will be investigated, the results may reveal a regulatory role for ROS.
